# SP1 tethers NR2F2 to drive atrial cardiomyocyte identity

**DOI:** 10.64898/2026.10.19.760167

**Published:** 2026-10-20

**Authors:** Payton N. Gergen, Alexander P. Clark, Jeffrey J. Saucerman

## Abstract

Human heart development is guided by complex gene regulatory networks (GRNs), which are difficult to unravel and differ from that in animal models. In this study, we use a systematic approach to identify gene interactions driving human fetal atrial cardiomyocyte identity. We inferred GRNs for atrial and ventricular cardiomyocytes from human fetal single-cell RNA-seq and ATAC-seq data, predicting transcription factors that differentially regulate atrial vs. ventricular cardiomyocyte identity. Comparison of GRNs constrained by ATAC-seq vs. promoter annotations revealed that during human heart development, the canonical transcription factor for atrial specification, NR2F2, drives atrial genes not directly but by interacting with SP1. The predicted NR2F2-SP1 relationship at atrial gene promoters was supported with AlphaFold predictions of biophysical tethering and comparison with previous ChIP-seq data. These predictions were experimentally validated in human iPSC-derived atrial cardiomyocyte differentiation, which was suppressed by SP1 knockdown. Further, proximity ligation experiments validated the physical interaction of NR2F2 and SP1 in the nucleus, coincident with atrial identity. This study reveals SP1 as a transcription factor necessary for atrial cardiomyocyte identity in human development, helping to coordinate the effects of NR2F2. Furthermore, comparing GRNs based on current and potential epigenetic state offers a novel systematic approach to study interactions between transcription factors, which is key to understanding complex GRNs.

